# Effects of postbiotic supplementation on growth performance, ruminal fermentation and microbial profile, blood metabolite and GHR, IGF-1 and MCT-1 gene expression in post-weaning lambs

**DOI:** 10.1186/s12917-019-2064-9

**Published:** 2019-09-02

**Authors:** Wan Ibrahim Izuddin, Teck Chwen Loh, Anjas Asmara Samsudin, Hooi Ling Foo, Ali Merzza Humam, Nurhazirah Shazali

**Affiliations:** 1 Department of Animal Science, Faculty of Agriculture, Universiti Putra Malaysia, 43400 Serdang, Selangor Malaysia; 2 Institute of Tropical Agriculture and Food Security, Universiti Putra Malaysia, 43400 Serdang, Selangor Malaysia; 3 Department of Bioprocess Technology, Faculty of Biotechnology and Biomolecular Sciences, Universiti Putra Malaysia, 43400 Serdang, Selangor Malaysia; 4 Institute of Bioscience, Universiti Putra Malaysia, 43400 Serdang, Selangor Malaysia; 5 Department of Animal Resources, Faculty of Agriculture, University of Baghdad, Baghdad, Iraq

**Keywords:** Postbiotics, *Lactobacillus plantarum*, Growth performance, Rumen fermentation, Blood metabolite, Gene expression, Nutrient uptake, Post-weaning lambs

## Abstract

**Background:**

Postbiotics have been established as potential feed additive to be used in monogastric such as poultry and swine to enhance health and growth performance. However, information on the postbiotics as feed additive in ruminants is very limited. The aim of this study was to elucidate the effects of supplementation of postbiotics in newly-weaned lambs on growth performance, digestibility, rumen fermentation characteristics and microbial population, blood metabolite and expression of genes related to growth and volatile fatty acid transport across the rumen epithelium.

**Results:**

Postbiotic supplementation increased weight gain, feed intake, nutrient intake and nutrient digestibility of the lambs. No effect on ruminal pH and total VFA, whereas butyrate and ruminal ammonia-N concentration were improved. The lambs fed with postbiotics had higher blood total protein, urea nitrogen and glucose. However, no difference was observed in blood triglycerides and cholesterol levels. Postbiotics increased the population of fibre degrading bacteria but decreased total protozoa and methanogens in rumen. Postbiotics increased the mRNA expression of hepatic IGF-1 and ruminal MCT-1.

**Conclusions:**

The inclusion of postbiotics from *L. plantarum* RG14 in newly-weaned lambs improved growth performance, nutrient intake and nutrient digestibility reflected from better rumen fermentation and microbial parameters, blood metabolites and upregulation of growth and nutrient intake genes in the post-weaning lambs.

## Background

In sheep production, weaning process is recognized as one of the stressful procedures which is influenced by environment, nutrition and psychology that potentially affect the growth performance and health of the lambs [1, 2]. Weaning stress contributes to a reduction in feed intake and therefore decrease growth rate and increase the vulnerability to diseases. These circumstances could affect the growth performance of lambs in the post-weaning period. Thus, good weaning management including nutritional modification is essential in alleviating the stress and improving health and performance at the post-weaning period. In ruminants, bacterial and yeast probiotics are being utilized as feed additives to generally enhance rumen fermentation and promote immune function and general health [3]. Probiotics in the rumen interact with rumen microbes to enhance rumen fermentation and synthesis of antimicrobial substances such as bacteriocins that inhibit the harmful pathogens in the gut [4]. Probiotic use in ruminants may contribute to beneficial effects in term of enhancing population of ruminal cellulolytic bacteria [5] leading to greater fibre digestibility, improving synthesis and bio-availability of nutrients contributing to a better growth performance [6]. The increment of feed intake, nutrient absorption, feed conversion ratio (FCR) leading to greater production performance [7, 8]. It has been reported that probiotics are used widely as feed supplement and may contributes better health and growth performance for the weaned lamb. However, probiotics are sensitive to environmental condition i.e. sun-light, pH of water. Thus, they need a proper and careful handling and offering to the weaned lamb. Consequently, it creates cumbersome to farmers in terms of application. Likewise, some probiotics may carry antibiotic-resistant genes, particularly bacteria with plasmid-encoded which able to transfer between organisms [9, 10]. The gene could transfer from probiotics to native microbes and potentially transfer to pathogens.

Thus, postbiotics are proposed to be an alternative as feed supplement due to ease of handling and application. Postbiotics are the metabolites of probiotic bacteria which are characterized by probiotic effect with the absence of living cells. Addition of postbiotics in the diets have been shown to improve growth performance in the monogastric animals such as broilers [11, 12], layers [13] and piglets [14, 15]. Presence of antimicrobial metabolites such as organic acids and bacteriocins in the postbiotics have been shown to exhibit high inhibitory activity against pathogenic bacteria such as *Salmonella typhimurium*, *Escherichia coli*, *Listeria monocytogenes*, *Pediococcus acidilactici* and Vancomycin-resistant Enterococci (VRE) [16, 17]. However, postbiotics as feed additive were not investigated in ruminants particularly on its effect on rumen fermentation and feed utilization efficiency. Thus, the objective of this study was to elucidate the effects of postbiotics from *Lactobacillus plantarum* RG14 on growth performance focusing on rumen fermentation characteristics and nutrient utilization in the post-weaning lambs. Therefore, the response of the postbiotics on rumen fermentation profiles and microbial population, nutrient intake and digestibility, blood metabolites and expression of genes related to growth and ruminal nutrients transporter were examined to improve the understanding of the postbiotic action in ruminants. The postbiotics from *L. plantarum* RG14 was chosen as this strain give highest cell population and inhibitory activity against several pathogenic microorganisms [18].

## Results

### Growth performance, nutrient intake and apparent digestibility

The postbiotic supplementation significantly increased (*P* < 0.05) final weight gain, total weight gain, average daily gain (ADG) (Table 1). No differences (*P* > 0.05) were found in total feed intake, daily feed intake and FCR.

**Table 1:** Growth performance characteristics in post-weaning lambs supplemented with and without postbiotics

Parameters	Control	Postbiotic	SEM	*P*-value
Initial weight (kg)	17.10	18.90	0.52	0.09
Final weight (kg)	25.60^a^	28.27^b^	0.65	0.03
Weight gain (kg)	8.35^a^	9.37^b^	0.27	0.04
ADG (g/d)	185.56^a^	208.15^b^	5.95	0.04
Total feed intake (kg DMI)	30.02	31.13	0.35	0.13
Daily feed intake (kg DMI/d)	0.67	0.67	0.001	0.13
FCR (kg DMI/kg gain)	3.56	3.33	0.07	0.01

*ADG* Average daily gain, *DMI* Dry matter intake, *FCR* Feed conversion ratio. ^a,b^ Different superscripts in each row are significantly different (*P* < 0.05). Experimental unit, (*n* = 6)

### Nutrient intake and apparent digestibility

The inclusion of postbiotics increased (*P* < 0.05) the DM, OM and NDF intake of the lambs (Table 2). No differences (*P* > 0.05) in the EE and CP intake of lambs. In terms of nutrient digestibility, lambs received postbiotics had higher (*P* < 0.05) digestibility of DM, CP and NDF. No differences (*P* > 0.05) in the OM and EE digestibility between two groups.

**Table 2:** Nutrient intake and apparent digestibility in post-weaning lambs supplemented with and without postbiotics

Parameters	Control	Postbiotic	SEM	*P*-value
Nutrient intake (g DM/d)				
DM	481.30^a^	493.80^b^	3.21	0.03
OM	443.60^a^	461.40^b^	4.16	0.02
EE	17.89	18.27	0.15	0.25
CP	114.38	115.88	0.85	0.42
NDF	396.86^a^	408.60^b^	3.37	0.08
Apparent nutrient digestibility (g/kg DM)				
DM	635.57^a^	719.03^a^	2.09	0.04
OM	729.56	776.00	1.36	0.09
EE	46.61	48.27	1.45	0.62
CP	673.48^a^	733.27^a^	1.35	0.01
NDF	386.11^a^	432.69^a^	0.99	0.01

*DM* Dry matter, *OM* Organic matter, *EE* Ether extract, *CP* Crude protein, *NDF* Neutral detergent fibre. ^a,b^ Different superscripts in each row are significantly different (*P* < 0.05). Experimental unit, (*n* = 6)

### Rumen fermentation characteristics

No differences (*P* > 0.05) in pH, total and individual VFA except in the propionic acid in which postbiotic inclusion increased (*P* < 0.05) the concentration of the propionic acid in the rumen fluid (Table 3). Concentration of ruminal NH_3_-N significantly increased (*P* < 0.05) in the postbiotic group compared to the control group.

**Table 3:** Rumen fermentation characteristics in post-weaning lambs supplemented with and without postbiotics

Parameters	Control	Postbiotic	SEM	*P*-value
pH	5.55	5.53	0.03	0.82
NH_3_-N, (ppm)	1.46^a^	2.00^b^	0.14	0.04
Volatile fatty acids (VFA), mM				
Acetic acid (A)	84.82	82.70	2.15	0.63
Propionic acid (P)	31.81^a^	37.56^b^	1.65	0.03
Butyric acid	9.30	9.31	0.37	1.00
iso-butyric acid	1.28	1.35	0.04	0.37
Valeric acid	1.18	1.15	0.06	0.89
iso-valeric acid	2.55	2.60	0.15	0.86
Total VFA	130.98	134.64	2.00	0.40
A:P	2.43	2.17	0.09	0.17

*VFA* Volatile fatty acids. ^a,b^ Different superscripts in each row are significantly different (*P* < 0.05). Experimental unit, (*n* = 6)

### Blood metabolites

The lambs received postbiotic supplementation in the diet had higher (*P* < 0.05) total protein, urea nitrogen and glucose concentration in the blood (Table 4). No significant differences (*P* > 0.05) were recorded in blood triglycerides and cholesterol in both groups.

**Table 4:** Blood metabolites in post-weaning lambs supplemented with and without postbiotics

Parameters	Control	Postbiotic	SEM	*P*-value
Total protein (g/L)	63.60^a^	98.97^b^	7.16	0.01
BUN (mmol/L)	6.03^a^	10.04^b^	0.88	0.01
Glucose (mmol/L)	4.47^a^	6.05^b^	0.34	0.01
Triglycerides (mmol/L)	0.30	0.43	0.06	0.34
Cholesterol (mmol/L)	1.83	1.60	0.01	0.28

*BUN* Blood urea nitrogen. ^a,b^ Different superscripts in each row are significantly different (*P* < 0.05). Experimental unit, (*n* = 6)

### Rumen microbial population

There was no significant difference (*P* > 0.05) in ruminal total bacteria in the lambs fed with and without postbiotics (Table 5). In the lambs receiving postbiotics, the population of two major fibre degrading bacteria which are *F. succinogenes* and *R. flavefaciens* were higher (*P* < 0.05) but no difference (*P* > 0.05) in population of *R. albus*. No difference (*P* > 0.05) in the population of protozoa between control and postbiotic groups. The reduction (*P* < 0.05) of population of the methanogens was observed in lambs receiving supplementary postbiotics in the feed.

**Table 5:** Ruminal microbial population (log_10_ microbial/mL) in post-weaning lambs supplemented with and without postbiotics

Parameters	Control	Postbiotic	SEM	*P*-value
Total bacteria	10.62	10.96	0.10	0.09
*F. succinogenes*	4.57^a^	5.81^b^	0.32	0.04
*R. flavefaciens*	11.58^a^	13.06^b^	0.30	0.01
*R. albus*	10.78	11.08	0.62	0.83
Protozoa	6.52	5.58	0.26	0.07
Methanogens	10.76^a^	10.03^b^	0.12	0.03

^a,b^ Different superscripts in each row are significantly different (*P* < 0.05). Experimental unit, (*n* = 6)

### Gene expression

**Fig. 1:**
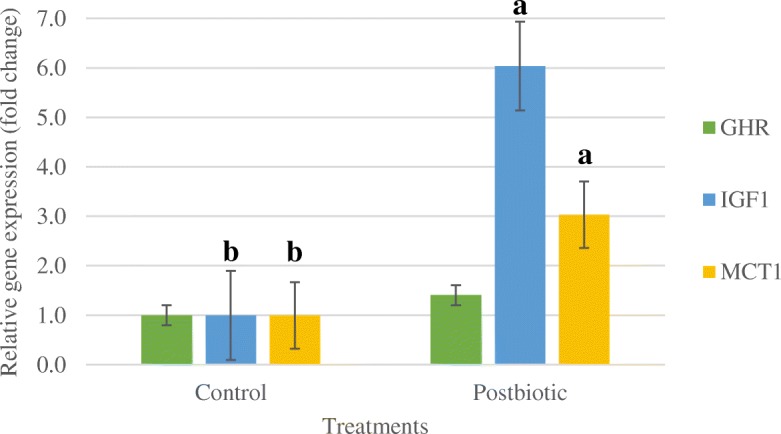
The GHR, IGF-1 and MCT-1 gene expression in post-weaning lambs fed with and without postbiotics. GHR Growth hormone receptor; IGF-1 Insulin-like growth factor 1; MCT-1 Monocarboxylate transporter 1. ^a,b^ Bar with different letter significantly differ (*P* < 0.05). Experimental unit, (*n* = 6)

No significant difference (*P* > 0.05) was observed in the expression of GHR gene between two groups (Fig. 1). Inclusion of postbiotics in the diet of lambs highly upregulated (*P* < 0.05) the hepatic expression of IGF-1 and ruminal expression of MCT-1 genes.

## Discussion

### Growth performance, nutrient intake and apparent digestibility

Weaning stress at the early life of production may arise from the changes in environment, social, nutrient and psychology which potentially compromise productivity, health and welfare [2]. Supplementation of postbiotics in the diet of post-weaning lambs enhanced the growth performance by the improvement in final body weight, body weight gain and ADG. Saleem et al. [19] reported that post-weaning lambs supplemented with probiotics had better growth performance in term of final weight gain, total gain, ADG and FCR. Better growth performance in lambs receiving postbiotics was accompanied by the increase in nutrient intake and digestibility of the feed which increase the availability of nutrients to the lambs. Addition of postbiotics in the diet improved nutrient digestibility of DM, CP and NDF. This finding is coherent with Saleem et al. [19] who reported that lambs supplemented with probiotic bacteria improved the digestibility of DM, CP, crude fibre and nitrogen-free extract. Postbiotic effect in term of promoting dry matter intake and improving fibre degradability is similar to the mode of action of probiotic bacteria. This may be explained by the improvement of the concentration of cellulolytic bacteria in the lambs supplemented with probiotics in the diet [20]. The increase in the rumen microbes will increase in the synthesis of microbial protein and provide higher amino acid supply to post-ruminal which help lambs to gain greater body weight [21].

In the present study, the increase in nutrient digestibility and intake due to postbiotic supplementation could provide greater protein and metabolizable energy to the lambs leading to better growth performance. In addition, the presence of antimicrobial compounds in postbiotics such as bacteriocins and organic acids exhibit inhibitory effect against various pathogens [16, 17] in the gastrointestinal tract providing better nutrient digestibility and absorption by the lambs. Galina et al. [22] found that the inclusion of a mixture of probiotic *Lactobacillus* in growing goats improved fibre and protein digestibility and enhanced microbial synthesis. Thanh et al. [16] reported that postbiotic inclusion in the diet of broilers decreased the proliferation of pathogenic bacteria which may ultimately improve feed utilization and thereby animal performance.

### Rumen fermentation characteristics

The value of rumen pH recorded in the present study was low considering higher concentrate ratio in the diet. The pH lower than 5.5 is prone to subacute rumen acidosis but with no clinical signs until the pH is below 5 which is considered acute or clinical acidosis [23]. Inclusion of postbiotics in the diet of lambs did not change the ruminal pH. It was consistent to the finding by Izuddin et al. [24] who reported postbiotic inclusion had no effect on rumen fluid pH in vitro. This finding was also observed by Vosooghi-Poostindoz et al. [25] who found no differences in pH in the lambs receiving probiotics at 16.5% CP in the diet. Although the pH of the postbiotics is low (pH 3.8–4.3), supplementation of 0.9% of postbiotics in the diet did not alter the rumen pH. No changes in rumen pH may suggest the adaptation and regulation of rumen environment to the presence of lactic acid from supplementation of postbiotics in the diet.

Postbiotics in the diet of lambs increased ruminal NH_3_-N concentration. Production of ammonia in the rumen is resulted from digestion of dietary protein and non-protein nitrogen by ruminal microorganism. Ammonia is crucial as a source of nitrogen for the growth of ruminal microorganisms and synthesis of microbial proteins. The major factors that influence the ruminal ammonia concentration are the amount of protein in the diet and the degradability of the protein in the rumen. The increase in degradability of protein in the rumen increases the ruminal concentration of ammonia. In the current study, the higher NH_3_-N concentration in the postbiotic diet was in line with the higher crude protein digestibility in lambs.

Supplementation of postbiotics in in vitro rumen fermentation has shown the increase in concentration of total and major individual VFA such as acetic, propionic and butyric [24]. However, postbiotic inclusion in post-weaning lambs did not affect total and individual VFA except for propionic acid. The finding was consistent with Astuti et al. [26] who reported the use of *L. plantarum* as probiotics in in vitro rumen fermentation study showed no effect on total VFA production. The finding was also consistent with Qadis et al. [27] who found no effect of probiotics from *L. plantarum*, *Enterococcus faecium* and *Clostridium butyricum* on total VFA in rumen fluid. The presence of high portion of lactic acid in the postbiotics provides a constant supply of lactic acid that might stimulate the lactic acid utilizing bacteria. Lactic acid utilizing bacteria such as *Propionibacterium spp.* produce more propionic acid rather than lactic acid [3] that may contribute to the greater molarity of propionic acid in the rumen fluid of lambs supplemented with postbiotics.

### Blood metabolites

Lambs received postbiotics in the diet had higher blood level of total protein, BUN and glucose. The *L. plantarum* generates certain carbohydrates into simpler compounds like glucose, which provides energy [28]. Higher blood glucose level in lambs supplemented with postbiotics might be associated with higher production and absorption of propionic acid as it is the precursor of glucose that enhances glucose production [29]. Likewise, higher population of *Propionibacterium* present in the rumen particularly in average concentrate diet alter the conditions in the rumen through utilization and conversion of lactic acid into propionic acid resulting in higher creation of hepatic glucose [30].

Blood total protein and BUN concentration can be a valuable indicator of the protein status in animals. Higher blood total protein and BUN concentration may be due to higher microbial digestibility producing high ammonia causing incapacity of ruminal microflora to optimally detain the ammonia [31]. The outcome was contrary to Saleem et al. [19] who found probiotic supplementation in post-weaning lambs has no effect on blood total protein and decreased blood urea. The discrepancy in these findings could be due to different capacity of nutrient digestibility or nitrogen utilization in the rumen which is reflected in the blood. No changes were detected in the serum level of triglycerides and cholesterol in control and postbiotic treatments. It can be related to the fact that no effect of postbiotics on crude fat digestibility and absorption of products of fat digestion by the small intestine. This is consistent with the findings of Vosooghi-Poostindoz et al. [25] who reported no difference in blood concentration of triglyceride and cholesterol in post-weaning lambs supplemented with probiotics at 16.5% CP in the diet.

### Rumen microbial population

The population of total bacteria, *R. albus* and total protozoa were not affected in both groups. The inclusion of postbiotics increased the population of cellulolytic bacteria, *F. succinogenes* and *R. flavefaciens* in the rumen. Izuddin et al. [24] reported the supplementation of postbiotics from *L. plantarum* RG14 in in vitro rumen fermentation increased total bacteria, total protozoa and major cellulolytic bacteria. The mechanisms of probiotic LAB in the rumen in modulating and interacting with rumen microorganisms are still unclear. Weinberg et al. [4] suggested the benefits of probiotic LAB on the improvement of rumen fermentation are due to the interaction of probiotic LAB and rumen microorganisms and inhibition of detrimental microorganisms by the presence of a variety of antimicrobial compounds such as bacteriocins. Similar to probiotic LAB, the presence of the lactic acid in postbiotics may enhance the adaptation of rumen microorganisms to lactic acid or impede the accumulation of lactic acid by anaerobic degradation of lactic acid to acetic acid as suggested by Ghorbani et al. [32] and Nocek et al. [33] which these conditions would promote the cellulolytic activities and improve degradation of fibres [34]. The cellulolytic bacteria such as *F. succinogenes* and *R. flavefaciens* increased with postbiotic supplementation may explain the improvements in cellulolytic activity leading to better digestion of DM and NDF of feed.

The lower ruminal population of methanogens was discovered following supplementation of postbiotics in lambs. Higher synthesis of propionate in the postbiotic group indicates the reduction of methane formation and increase in retention of energy from the diet [35]. Production of propionate is involving in H_2_-utilisation pathway and as H_2_ is the main precursor of methane formation, decreased in the availability of H_2_ is associated with a decrease in methane production [35, 36]. This condition may reflect the reduction of ruminal methanogens population in the postbiotic group as the depletion of H_2_ as the source of methane production by methanogens.

### Gene expression

In the current study, post-weaning lambs received postbiotics had higher regulation of hepatic IGF-1 mRNA by 5 folds compared to control. The IGF-1 is the mediator of pituitary growth hormone which is synthesized and secreted in the liver to stimulate cell growth. The increase in expression of IGF-1 reflected in the growth performance of lambs supplemented with postbiotics. Mears [37] reported that plasma IGF-1 concentration in lambs at an early age has a positive correlation with the growth rates and it could be a useful indicator of growth potential and aid in the selection of fast-growing animals. Similar finding was reported as postbiotic supplementation in the diet of broilers increased the growth performance of birds and enhanced the fold expression of IGF-1 and GHR mRNA [12].

Postbiotic inclusion in the diet of post-weaning lambs upregulated the MCT-1 mRNA expression in the rumen tissue. Higher expression of MCT-1 gene can be associated with higher uptake of VFA and their metabolites through rumen epithelium into the blood to be metabolized to generate energy. Kuzinski and Röntgen [38] reported the elevated level of butyric acid in the rumen of lambs shifted from hay diet to hay and concentrate diet responsible for the upregulation of MCT-1 mRNA. However, in the current study, no differences were observed in butyric acid concentration in rumen fluid between control and postbiotic groups. Besides nutritional induction, MCT-1 gene can be upregulated by the synthetic peroxisome proliferator-activated receptor α (PPAR-α) agonists which is a transcription factor that mediates the adaptive response to fasting [39]. In response to feeding and starvation, PPAR-α acts as nutritional sensor which permits adaptation of the rates of fatty acid catabolism, lipogenesis and ketone body synthesis [40, 41]. Dijkstra et al. [42] reported that the rate of absorption of VFA through rumen epithelium linearly increased with the decreasing ruminal pH. The decline in the pH will provide a higher proportion of VFA in the undissociated form which is more permeable across the lipid bilayer of the cell wall [43]. In the present study, slightly lower pH of rumen fluid in the postbiotic group can contribute to a greater uptake of the VFA.

## Conclusions

The findings from the current study showed that the inclusion of 0.9% postbiotics from *L. plantarum* RG14 improved growth performance, nutrient intake and nutrient digestibility in the diet of post-weaning lambs. The improvement of growth and feed utilization in lambs fed with postbiotics were reflected by the rumen fermentation characteristics, ruminal cellulolytic bacteria population and blood metabolites. The higher expression of hepatic IGF-1 and ruminal MCT-1 mRNA resulted in higher production of IGF-1 in the liver and greater uptake of VFA through the ruminal epithelium, respectively. Postbiotics are the potential feed additive to be used to promote rumen fermentation and growth of post-weaning ruminant animals. We extrapolate the effects of postbiotics would be similar in different sexes of lambs, however the effects of postbiotics on growth and reproductive performance in female lambs are worth to be explored in the near future.

## Methods

### Microorganisms and maintenance

The *L. plantarum* RG14 was obtained from the Laboratory of Industrial Biotechnology, Department of Bioprocess Technology, Faculty of Biotechnology and Biomolecular Sciences, Universiti Putra Malaysia. The bacterial cultures was maintained and revived as described by Foo et al. [44] and Moghadam et al. [45]. The bacterial cultures were maintained at − 20 °C in de Man, Rogosa and Sharpe (MRS) medium (Merck, Germany) supplemented with 20% (v/v) glycerol.

### Postbiotic production from *L. plantarum* RG14

The active *L. plantarum* RG14 was washed once with sterile 0.85% (w/v) NaCl (Merck, Germany) solution and adjusted to 10^9^ CFU/mL to be used as inoculum. The working cultures of *L. plantarum* RG14 was prepared by inoculating 10% (v/w) of 10^9^ CFU/mL active bacterial cell into MRS media and incubated at 30 °C for 10 h, followed by centrifugation (Benchtop Microfuge 20R, Beckman Coulter, Germany) at 10,000×g, 4 °C for 15 min. The cell free supernatant (CFS) was then collected by filtration through a cellulose acetate membrane (Sartorius Minisart, 0.22 μm, Germany) as described by Loh et al. [46]. The CFS was stored at − 20 °C until feeding trial was conducted.

### Animals and management

The study was conducted in the Department of Animal Science Research Farm, Universiti Putra Malaysia. A total of twelve healthy newly-weaned, gonadally intact male Dorper lambs at 112 days of age with an average body weight of 17.3 ± 0.58 kg (at the beginning of adaptation period) were randomly allocated to two treatment groups. The control group received no postbiotics and the other group received 0.9% (v/w) postbiotics in the diet. The level of inclusion was based on the in vitro study reported by Izuddin et al. [24]. The lambs were individually housed in slatted floor pen (1 m × 1.5 m) and offered the isocaloric and isonitrogenous diet for 60 days including 14 days of adaptation period. The diets were formulated according to nutritional requirements of sheep by NRC [47] using FeedLIVE software (Thailand). The ingredients and chemical composition of the diets are presented in Table 6. The amount of grass and concentrate offered was adjusted weekly based on the 4 % of body weight of that particular week. Grass was given in the morning at 08:00 and concentrate was added in the evening at 16:00 daily. The daily amount of individual lamb intake of grass was considered during experimental period to ensure the concentrate given meet the grass to concentrate ratio. Fresh drinking water was continuously provided in each pen.

**Table 6:** Feed composition and nutrient content of the feed

	Control	Postbiotic
Feed composition (%)		
Grass	30.00	30.00
Corn	40.00	40.00
Soybean	23.80	23.80
Wheat pollard	3.40	3.40
Crude palm oil	0.90	0.90
Calcium carbonate	1.70	1.70
Salt	0.40	0.40
Mineral premix^a^	0.90	0.90
Vitamin premix^b^	0.90	0.90
Postbiotic RG14	–	0.90
Chemical nutrient composition (g/kg DM)		
Crude protein	169.00	169.00
Crude fat	27.00	26.70
NDF	596.00	596.00
ADF	169.00	166.00

^a^Mineral mix contains Co 0.6 mg, Cu 20 mg, Fe 100 mg, I 2 mg, Mn 110 mg, Se 0.2 mg, Zn 100 mg

^b^Vitamin premix contains vitamin A 0.45 mIU/kg, vitamin B1 0.09 g/kg, vitamin B2 0.27 g/kg, vitamin B6 0.18 g/kg, vitamin B12 0.09 mg/kg, vitamin D3 0.09 mIU/kg, vitamin E 0.67 g/kg, vitamin K3 0.18 g/kg, biotin 2.12 mg/kg of feed

*NDF* Neutral detergent fibre, *ADF* Acid detergent fibre

The diets were formulated using feed live international software (Thailand)

### Data, sample collection and analysis

Body weight of the lambs was recorded before the morning feeding at the beginning of the feeding trial and on weekly basis throughout the feeding trial. Feed intake was determined daily by measuring the difference in the amount of feed offered and refusal. The moisture content of the feed offered and refused was considered on daily basis to correct dry matter intake of the feed. During the experimental period, the ratio of the grass to concentrate was maintained at the 30:70. Feed conversion ratio (FCR) was calculated for each individual lamb from total feed intake and total weight gain during the experimental period. In determination of nutrient digestibility, daily feed and faecal collection was done for seven consecutive days at the final week of the feeding trial in the morning. Daily total faecal voided was weighed and 10% (w/w) of the total faecal was sampled and kept frozen at − 20 °C. At the end of the collection period, faecal samples of the individual lamb for 7 days were composited and subsampled for further analysis. Blood samples were collected by jugular venepuncture on day 58 of the feeding trial period before morning feeding into the BD Vacutainer® serum tube.

At day 60 of the feeding trial, all lambs were transferred to the research abattoir in the Department of Animal Science, Universiti Putra Malaysia to fast overnight in the lairage with access to fresh drinking water. The following day, the lambs were sacrificed without stunning according to the Halal slaughtering procedure (MS1500: 2009) by severing the carotid artery and jugular vein as outlined by Malaysian Standard [48]. After slaughtering, lambs were eviscerated for the collection of rumen fluid, rumen and liver tissues. Rumen fluid was collected for the analysis of pH, ammonia-N (NH_3_-N) concentration, VFA and microbial population. Ruminal pH was measured shortly after rumen fluid collection from rumen using a pH meter (Mettler-Toledo Ltd., England, UK). Approximately 2 cm^2^ of rumen tissues from the caudal dorsal of rumen and small portion of liver from left lobe of liver were collected and immediately frozen in liquid nitrogen and kept in − 80 °C freezer upon extraction of RNA.

### Proximate analysis and apparent digestibility

The DM content was determined by drying at 60 °C for 24 h. Analytical DM of the oven-dried samples was determined by drying at 105 °C for 24 h. Ash content was determined by combustion of samples in 550 °C muffle furnace for 5 h. Nitrogen content was determined by the Kjeldahl method using Foss Digestor™ System for sample digestion and Foss Kjeltec 2300 for distillation and titration [49]. Crude fat was determined by ether extract method using the Foss Tecator Soxtec 2050 Avanti (FOSS, Denmark). Contents of NDF and ADF were determined by neutral detergent and acid detergent solutions respectively following the methods detailed by Van Soest et al. [50]. Feed and faecal samples were analysed for DM, OM, CP, EE and NDF for the determination of apparent digestibility.

### Blood metabolites

The serum was separated and collected by centrifugation at 3000 rpm for 5 min for the determination of blood metabolites. The analysis of glucose, total protein, urea nitrogen, triglyceride and cholesterol levels in the serum were determined by Hitachi 902 Automatic Analyser (Roche Diagnostics, Germany) using appropriate kits.

### Ruminal NH_3_-N and VFA

Thawed rumen fluid samples were centrifuged for 6000×g for 10 min at room temperature. Supernatant collected were analysed for NH_3_-N based on the protocol described by Parsons et al. [51]. The absorbance of the colour intensity was measured at 640 nm by using Spectrophotometer (GENESYS™ 20 Thermo Scientific™, USA). The concentration of NH_3_-N was determined by constructing a standard curve of known concentration and substituting the absorbance of the sample in the standard curve equation. For the analysis of VFA, rumen fluid was acidified with 25% metaphosphoric acid (w/v) in the ratio of 4:1 (v/v) respectively and centrifuged at 3000×g for 10 min. The supernatant was collected, filtered and used for VFA determination. The 4-methyl-n-valeric acid (Sigma, St. Louis, MO) was used as an internal standard. Then, 0.5 mL of clear supernatant was collected and mixed with equal volume of 4-methyl-valeric acid (Sigma-Aldrich, St. Louis, MO) as internal standard. The VFA of the rumen fluid were analysed using a 6890 N Network GC System gas chromatograph (Agilent Technologies) according to Filípek and Dvořák [52]. Separation of VFA profile was determined using Quadrex 007 Series (Quadrex Corp., New Haven, CT 06525, USA) bonded phase fused silica capillary column (15 m, 0.250 mm ID, 0.25 μm film thickness) with a 6890 N Network GC System gas chromatograph (Agilent Technologies) equipped with a flame ionisation detector. Nitrogen gas was supplied as carrier gas at the rate of 60 mL/min. The temperature of the column was set at 200 °C and injector and detector both at 230 °C. Commercial standards (Sigma-Aldrich, St. Louis, MO) of 20 mM acetic, and 10 mM each of propionic, butyric and 4-methyl-valeric acids were used as external standards for peak identification. Molar concentration of VFA were identified based on single point of internal and external standards.

### Rumen microbial population

The isolation of microbial DNA in rumen fluid using QIAamp® Fast DNA Stool Mini Kit (Qiagen, Hilden, Germany), following the manufacturer’s protocol. The DNA concentration was measured using Nanodrop 2000 spectrophotometer (Thermo Scientific, Wilmington, DE). Only high DNA concentration of higher than 30 ng/μL and high purity was chosen for further manipulation. Absolute quantification of microbes in the rumen fluid was performed based on the standard curve of amplification of target microbes. The populations of total bacteria, *Ruminococcus albus*, *Ruminococcus flavefaciens*, *Fibrobacter succinogenes*, methanogens and protozoa were analysed by using qPCR. The targeted microbes, the sequences of the forward and reverse primers and the annealing temperature are shown in Table 7. Real-time qPCR was performed with the Bio-Rad CFX96 Real-time PCR system (Bio-Rad Laboratories, CA, USA). The qPCR cycling condition consisting of initial heat activation at 95 °C for 10 min, following by 40 cycles of denaturation at 95 °C for 15 s, annealing at 55 °C for total bacteria, *F. succinogenes*, *R. albus* and protozoa, 58 °C for methanogens and 60 °C for *R. flavefaciens* for 30 s and finally 30 s of extension at 72 °C. The analysis of melting curve was performed at the end of the amplification cycle to confirm the specificity of amplification.

**Table 7:** Sequence of polymerase chain reaction primers used to target microbes

Target microbes	Primer	References
Total bacteria	F - CGGCAACGAGCGCAACCC R - CCATTGTAGCACGTGTGTTAGCC	[53]
*F. succinogenes*	F - GTTCGGAATTACTGGGCGTAAA R - CGCCTGCCCCTGAACTATC	[54]
*R. albus*	F - CCCTAAAAGCAGTCTTAGTTCG R - CCTCCTTGCGGTTAGAACA	[55]
*R. flavefaciens*	F - TCTGGAAACGGATGGTA R - CCTTTAAGACAGGAGTTTACAA	[55]
Methanogens	F - TTCGGTGGATCDCARAGRGC R - GBARGTCGWAWCCGTAGAATC	[56]
Protozoa	F - CTTGCCCTCYAATCGTWCT R - GCTTTCGWTGGTAGTGTATT	[57]

*F* Forward, *R* Reverse

### RNA extraction and RT-PCR of GHR, IGF-1 and MCT-1 genes

The tissues were pulverized by mortar and pestle with presence of liquid nitrogen. Total RNA was isolated from liver and rumen tissues samples using RNeasy® Mini Kit (Qiagen, Hilden, Germany), following the manufacturer’s protocol. The concentration and purity (260/280 nm ratio of absorbance readings) of RNA were quantified by Nanodrop 2000 spectrophotometer (Thermo Scientific, Wilmington, DE). Approximately 100 ng/μL purified RNA was converted into complementary DNA (cDNA) using Quantitect® reverse transcription kit (Qiagen, Hilden, Germany) following the manufacturer’s procedure. The genomic DNA removal was performed before the reverse transcription of RNA to cDNA. Real-time qPCR was performed with the Bio-Rad CFX96 Real-time PCR system (Bio-Rad Laboratories, CA, USA). The targeted genes, the sequences of primers, product size are described in Table 8. The qPCR cycling condition consisting of initial heat activation at 95 °C for 10 min, following by 40 cycles of denaturation at 95 °C for 15 s, annealing at 57 °C for GADPH and GHR genes, 60 °C for MCT-1 gene for 30 s and finally 30 s of extension at 72 °C. The analysis of melting curve was performed at the end of the amplification cycle to confirm the specificity of amplification. The annealing temperature of target and reference genes were determined by the gradient protocol of Bio-Rad CFX96 Real-time PCR System (Bio-Rad Laboratories, CA, USA). The relative expression of the gene was measured according to the Livak’s method of 2^-ΔΔCt^ (ΔΔCt = ΔCt treated sample - ΔCt control sample) as described by Livak and Schmittgen [58]. For the internal standard (housekeeping gene), GADPH gene was used as to standardize the expression. The efficiency of amplification of target and housekeeping genes was determined by a 5-fold serial dilution of cDNA as a standard curve. All the standard curves showed good PCR amplification efficiencies between 90 to 120%.

**Table 8:** Primer information of target and reference genes

Target gene	Primer Sequence (5′ - 3′)	Product size (bp)	NCBI accession number
GHR	F - GCCAAAACAATAAGACTGGGAACC R - GGCTGTAGTGGTAAGGCTTTCTGTG	218	XM_012096676.1
IGF-1	F - ATTACAGCTGCCTGCCCCTT R - CACATCTGCTTACACCTTACCCG	265	NM_001009774.3
MCT-1	F - TGGCATCTTATCAGGCAGTGG R - CCAGCCACACAGCAGTTTAATAG	300	XM_004002335.3
GADPH	F - ACCACTTTGGCATCGTGGAG R - GGGCCATCCACAGTCTTCTG	76	NM_001190390.1

*F* Forward, *R* Reverse, *GHR* Growth hormone receptor, *IGF-1* Insulin-like growth factor 1, *MCT-1* Monocarboxylate transporter 1, *GADPH* Glyceraldehyde 3-phosphate dehydrogenase

### Statistical analysis

The experiment was subjected to a completely randomized design. For each parameter, the individual lamb was considered as one experimental unit. The differences between treatments were analysed using independent t-test. The initial body weight of lambs was used as covariate and it was analysed using analysis of covariance. The statistical software of SAS (Statistical Analysis System) software version 9.2 (SAS Institute, USA) was used to analyse the data. Differences between treatment means were considered significant at *P* < 0.05.

## Data Availability

The datasets used and/or analysed during the current study are available from the corresponding author on reasonable request.

## Abbreviations

ADG: Average daily gain
BUN: Blood urea nitrogen
CP: Crude protein
DM: Dry matter
EE: Ether extract
GHR: Growth hormone receptor
IGF-1: Insulin-like growth factor 1
LAB: Lactic acid bacteria
MCT-1: Monocarboxylate transporter 1
NDF: Neutral detergent fibre
NH_3_-N: Ammonia nitrogen
OM: Organic matter
VFA: Volatile fatty acid
